# Reducing Emotional Distress with Open-Label Placebos: Assessing the Role of Motor Engagement in Pill Consumption

**DOI:** 10.3390/bs14060455

**Published:** 2024-05-29

**Authors:** Anne Schienle, Wolfgang Kogler

**Affiliations:** Clinical Psychology, University of Graz, 8010 Graz, Austria

**Keywords:** open-label placebo pill, motor action, cognitive reappraisal, emotional distress

## Abstract

It has been posited that ingesting a pill constitutes a pivotal action that facilitates the effects of open-label placebos (OLPs: placebos honestly prescribed). In the present OLP experiment, the motor components of a placebo treatment were systematically varied. The participants (*n* = 183) were randomly allocated to one of four groups that all viewed aversive pictures. The ‘active OLP’ group took a placebo pill with specific instructions concerning the sequence of motor actions for the intake. The ‘usual OLP’ group swallowed the pill (without specific motor instructions), while the third group received an ‘imaginary OLP’ (no pill intake). The fourth group applied cognitive reappraisal (CR; active control group) to reduce emotional distress. The participants rated their affective state as well as the efficacy and plausibility of the treatment approach. Moreover, blood pressure and pulse were recorded as indicators of bodily arousal. The four groups did not differ in their valence ratings and physiological measures. The ‘imaginary OLP’ received higher ratings for both effectiveness and plausibility than the ‘usual OLP’. CR was rated as superior relative to all OLP conditions. In conclusion, reducing emotional distress with OLPs does not necessitate the consumption of a placebo pill. In terms of acceptability and ease of implementation, CR stands as a well-established alternative.

## 1. Introduction

Honestly administered placebos, commonly referred to as non-deceptive placebos or open-label placebos (OLPs), have exhibited effectiveness in mitigating symptoms across a wide spectrum of clinical conditions, including chronic back pain, irritable bowel syndrome, and attention-deficit/hyperactivity disorder (meta-analyses [1,2]; review [3]). Furthermore, investigations involving healthy participants have indicated that OLPs can reduce emotional distress, as indexed by both self-reports and neurobiological measures [4,5,6].

Some placebo researchers have suggested that the physical action of taking a pill is a key active component of an OLP condition ([7,8]; for a discussion see [9]). Therefore, an instruction that has been used in numerous OLP studies includes the following points: ‘(1) the placebo effect is powerful, (2) the body can automatically respond to taking placebo pills, like Pavlov’s dogs who salivated when they heard a bell, (3) a positive attitude helps but is not necessary, and (4) taking the pills faithfully is critical.’ ([10], p. 2). The last point underscores the crucial role of the embodied act of ingesting placebo pills in facilitating the placebo effect.

In medical contexts during pharmacotherapy, patients learn that a specific motor behavior (e.g., taking a pill) is associated with the reduction of their symptoms [11]. These learning experiences (conditioning) influence patients’ conscious and nonconscious expectations [12]. The motor actions prime the mind and body for healing by activating positive expectations and beliefs about the effectiveness of the treatment. Over time, the behavior of taking a pill develops symbolic meaning. The act of opening the pill package, placing the pill on the tongue, and swallowing it comprises sensory/motor elements that trigger distinct thoughts regarding the pill and its effects, such as “I am taking medication” and “I am treating my illness”. These assumptions align with the theory of embodied cognition, which suggests that cognitive processes are deeply intertwined with bodily experiences and perceptions [7,8,13].

To the best of our knowledge, motor aspects of OLP treatment have not yet been systematically investigated. So far, only two studies have compared the effects of conditions with and without the actual ingestion of a placebo pill [14,15]. In one study, healthy students reporting test anxiety were randomly assigned to one of three conditions: a 3-week intervention involving daily intake of an OLP pill, an imaginary pill, or a control condition (without a pill). Interestingly, both placebo interventions demonstrated similar efficacy in reducing test anxiety. In a functional magnetic resonance imaging study [15], participants were randomly allocated to one of three groups that all viewed repulsive images. The groups either passively viewed the images, swallowed an OLP pill, or received the following instruction: “The belief that a placebo works is crucial for the placebo effect. The psychological component is critical. Thus, from a logical standpoint, it is not necessary to take an inactive substance (placebo) to be able to reduce negative emotions. Therefore, imagine that you are being supported by a placebo”. (p. 2). This condition (‘imaginary OLP’) reduced disgust more effectively than the ingested OLP pill, as indexed by self-reports and brain activity. Furthermore, the imaginary OLP was perceived as a more plausible method to reduce emotional distress than the traditional OLP.

In the current experiment, participants were randomly assigned to either one of three OLP groups with varying levels of motor involvement or to an active control group (cognitive reappraisal; CR) before being presented with negative pictures. We decided to use an active control group because positive effects of OLPs relative to no treatment have been shown before (see meta-analysis by [16]). Nevertheless, to pave the way for the integration of OLPs into (clinical) practice, it is important to demonstrate their efficacy relative to the commonly used methods for alleviating emotional distress. CR is a well-established strategy that involves the cognitive reinterpretation of negative stimuli (see meta-analysis by [17]).

All participants of the present experiment received general information on non-deceptive placebos (OLP groups) or cognitive reappraisal (CR group) including previous research findings and assumed mechanisms. The ‘active OLP’ group then received a placebo pill with detailed instructions on the sequence of actions for oral administration (e.g., open the package, take out the blister pack, press the pill through the foil, place the pill on your tongue…). The ‘usual OLP’ group received an unpacked pill without detailed instructions (‘Please swallow this placebo pill’). The third OLP group was informed that from a logical standpoint, the oral intake of a pill without any active ingredients is not necessary for the placebo effect to occur. This group was asked to imagine having taken a placebo pill to reduce emotional distress (‘imaginary OLP’). Participants in the CR group were instructed to reassess the authenticity of what is being depicted (‘This is not real’).

After these group-specific instructions, all participants viewed unpleasant and neutral pictures, which were rated according to the arousal and valence elicited. During the experiment, blood pressure (BP) and pulse were recorded three times (baseline assessment, before picture viewing, and after picture viewing). BP and pulse were selected as the physiological indicators of bodily arousal during affective picture viewing [18]. It was predicted that the ‘active OLP’ would be more efficient in terms of reducing emotional distress and associated somatic arousal compared to the other two OLP interventions (usual, imaginary). Following an exploratory approach, it was investigated whether emotional distress and physiological measures would differ between the CR group and the three OLP groups. It was further analyzed whether the four groups would differ regarding the perceived efficacy and plausibility of the interventions.

## 2. Method and Materials

### 2.1. Participants

A total of 183 female participants (*M_age_* = 23.4 years, *SD* = 4.11; 98% with a high school diploma, 52% psychology students) were randomly allocated (random number table) to one of four groups: active OLP (*n* = 45), usual OLP (*n* = 45), imaginary OLP (*n* = 47), or CR (*n* = 46). The groups did not differ in mean age (*F*(3, 176) = 1.37, *p* = 0.25). Exclusion criteria were self-reported diagnoses of mental disorders, neurological disorders, psychotropic medication, male gender, and age < 18 years. The sample was restricted to females because of sex differences in emotional reactivity to unpleasant images [19]. Moreover, research on deceptive placebos has indicated that males and females differ in how they respond to verbal placebo suggestions [20]. Finally, ref. [21] suggested that sex differences in placebo responses could be due to sex differences in the underlying physiological processes mediating placebo effects.

We used G*power, version 3 [22] to estimate the sample size for the present study. To detect an effect size of 0.024 (partial *η*^2^) and achieve a power of 0.95 at an alpha level of 0.05 (four groups, two picture conditions, correlation among repeated measures = 0.5), a sample of 180 participants was required.

### 2.2. Procedure

This study with a parallel design (preregistration on the German Clinical Trials Register (DRKS00029212, June 7, 2022) (OLP groups registered; CR group added for exploratory comparison) was conducted between June 8, 2022 and October 1, 2022. Eligible participants (*n* = 183) were scheduled to come to the lab for an experiment on affective picture processing.

The participants first rated their affective state (valence, arousal) using a slider ranging from 0 to 100 (0 = I feel not unpleasant, not aroused; 100 = extremely unpleasant, aroused). Moreover, a baseline assessment for pulse and blood pressure (BP) was conducted (boso-medicus uno upper arm blood pressure system; Bosch + Sohn GmbH und Co. KG, Jungingen, Germany). Afterwards, participants were randomly allocated to one of the four groups (active OLP, usual OLP, imaginary OLP, CR).

The experiment continued with a visual presentation delivering general information on non-deceptive placebos in the OLP groups (active, usual, imaginary) or on cognitive reappraisal in the CR group. In the placebo presentation, it was stated that placebos are inert substances (e.g., sugar pills) or sham interventions that can help to reduce symptoms of certain conditions (e.g., negative emotions). The placebo response was defined as a mind–body response based on either learning experiences or positive expectations (instruction based on [10]. The presentation on cognitive reappraisal described CR as an emotion-regulating strategy involving the reinterpretation of negative stimuli or situations to reduce the intensity of negative emotions. Furthermore, it was stated that CR is associated with activity in the cognitive control areas of the brain, which can inhibit other brain regions linked to stress and negative emotions.

The general information was followed by specific instructions, which were delivered verbally in the form of a standardized protocol by the experimenter. The active OLP group received a placebo package with detailed verbal instructions on the sequence of actions for the oral administration of one placebo pill (1 cm long capsule filled with 0.8 g sugar). The participants were guided through each step of the administration process. They were asked to open the package, take out the blister pack, press the pill through the foil, put the pill on their tongue, take a sip of water, and swallow the pill. In the usual OLP group, participants were not guided through the sequence of actions for the pill’s administration (‘please swallow this placebo pill’; the pill was provided without the packaging). Both groups were informed that the pill was filled with sugar. The imaginary OLP group was asked to imagine taking the pill. (‘You have just been informed that placebos do not contain any active ingredients. So, if you look at it logically, you do not have to take the placebo for it to work. Please imagine that you would have taken a pill to reduce emotional distress’.). The CR group was instructed to apply cognitive reappraisal by imagining that the shown situations are not real (scenes from a movie).

After the specific instructions were given and the placebo was administered (for the active/usual OLP groups), the second physiological measurement (pulse, blood pressure) was conducted, and the participants were asked to evaluate the expected efficacy of the treatment to reduce emotional distress (1 = not effective; 9 = very effective). Subsequently, the participants viewed six unpleasant and six neutral images. The negative pictures (e.g., car accident, war scene) and the neutral pictures (everyday objects, such as dishes, bookshelf) were taken from the International Affective Picture System [23]; picture numbers: 0004, 2683, 3550, 6550, 6838, 9910, 5395, 7100) and a validated picture set by the authors. The pictures were presented in a randomized order. In each trial, participants first viewed a fixation cross (1000 ms) and then a picture (6000 ms). After each picture, the participants rated how the picture made them feel on a scale ranging from 0 to 100 (0 = I felt not unpleasant, not aroused; 100 = extremely unpleasant, aroused). Blood pressure and pulse were recorded again after they viewed the picture.

**Figure 1:**
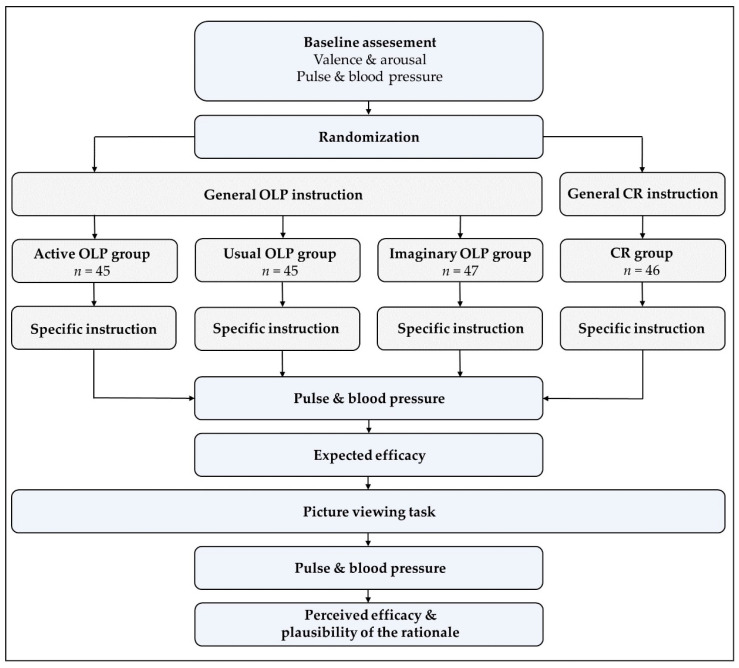
Procedure.

At the end of the experiment, the participants rated the perceived efficacy of the intervention (1 = not effective; 9 = very effective) as well as the plausibility of the treatment rationale via a slider (1 = not plausible; 9 = very plausible). Figure 1 depicts a flow chart of the experimental procedure. (The CONSORT flow diagram can be found in Supplementary Materials S1). The methods were not changed after the commencement of the trial. All participants completed the experiment (no dropouts).

### 2.3. Statistical Analyses

Mixed-effects analyses of variance (ANOVAs) tested the effects of Group (active OLP, usual OLP, imaginary OLP, CR) and Picture (negative, neutral) on ratings for valence and arousal elicited by the images. Ratings for the efficacy of the intervention (expected, perceived) and plausibility were compared between the groups via ANOVAs. Additional ANOVAs were computed to investigate the effects of Group and Time (baseline assessment, before picture viewing, after picture viewing) on the physiological measures (pulse, systolic/diastolic blood pressure). Given the reasonable robustness against normality violations, ANOVAs were computed despite some deviations from homoscedasticity and normality in the data. We report partial eta squared and Cohen’s d as the effect size measures. Post hoc *t*-tests were Holm-corrected.

## 3. Results

### 3.1. Baseline: Valence and Arousal

The four groups did not differ in their affective state (valence, arousal) before viewing of the pictures was carried out (valence: *F*(3, 179) = 0.005, *p* = 0.99, *η*^2^ < 0.001; arousal: *F*(3, 179) = 1.16, *p* = 0.33; *η*^2^ = 0.02; see Table 1).

### 3.2. Picture Viewing: Valence and Arousal

The ANOVA for valence ratings revealed a significant main effect for Picture (*F*(1, 179) = 379.68, *p* < 0.001, *η_p_*^2^ = 0.68). Negative pictures elicited greater unpleasantness than neutral pictures (Table 1). The effects for Group (*F*(3, 179) = 2.33, *p* = 0.08, *η_p_*^2^ = 0.04) and the Group x Picture interaction (*F*(3, 179) = 1.45, *p* = 0.23, *η_p_*^2^ = 0.02) were not significant.

The ANOVA for the arousal ratings revealed a significant main effect for Picture (*F*(1, 179) = 256.30, *p* < 0.001, *η_p_*^2^ = 0.59). Participants reported higher arousal for negative pictures than for neutral pictures (Table 1). The effect for Group also reached statistical significance (*F*(3, 179) = 2.93, *p* = 0.04, *η_p_*^2^ = 0.05). Post hoc *t*-tests showed that arousal was rated lower in the CR group compared to the imaginary OLP group (*t*(179) = 2.85, *p* = 0.03, *d* = 0.68) (Table 1). There were no other significant group differences for arousal ratings (all *p* > 0.05). The Group x Picture interaction was not significant (*F*(3, 179) = 1.11, *p* = 0.35, *η_p_*^2^ = 0.02).

### 3.3. Pulse and Blood Pressure

The ANOVAs conducted for pulse and blood pressure (systolic, diastolic) did not reveal any statistically significant differences between groups (all *p* > 0.10). The detailed results for the physiological measures can be found in Supplementary Materials S2 (means/standard deviations) and Supplementary Materials S3 (ANOVAs).

### 3.4. Perceived Efficacy and Plausibility of the Treatment

**Figure 2:**
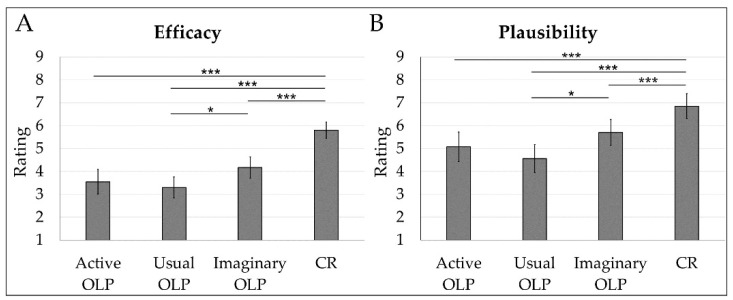
Mean ratings for efficacy (average of expected and perceived efficacy) in the four groups (**A**). Mean ratings for the plausibility of the rationale in the four groups (**B**). Error bars indicate 95% confidence intervals. * *p* < 0.05. *** *p* < 0.001.

The ANOVA for efficacy (expected, perceived) revealed a significant main effect for Group (*F*(3, 179) = 22.7, *p* < 0.001, *η_p_*^2^ = 0.28). Post hoc *t*-tests showed that the imaginary OLP group rated the intervention as more effective than the usual OLP group (*t*(179) = 2.63, *p* = 0.03, *d* = 0.54). Furthermore, efficacy was rated higher in the CR group compared to the three OLP groups (active: *t*(179) = 6.73, *p* < 0.001, *d* = 1.43; usual: *t*(179) = 7.48, *p* < 0.001, *d* = 1.77; imaginary: *t*(179) = 4.92, *p* < 0.001, *d* = 1.11) (Figure 2). Other post hoc comparisons between groups were not statistically significant (active OLP vs. usual OLP: *t*(179) = 0.75, *p* = 0.46, *d* = 0.15; active OLP vs. imaginary OLP: *t*(179) = 1.87, *p* = 0.13, *d* = 0.36). The effect for Time (*F*(1, 179) = 3.69, *p* = 0.06, *η*^2^ *=* 0.02) and the Group x Time interaction (*F*(3, 179) = 0.68, *p* = 0.57, *η*^2^ *=* 0.01) were not significant.

The ANOVA for the plausibility of the treatment rationale was significant (*F*(3, 179) = 10.5, *p* = <0.001, *η*^2^ = 0.15). Post hoc *t*-tests showed that the imaginary OLP group registered higher ratings than the usual OLP group (*t*(179) = 2.66, *p* = 0.03, *d* = 0.56). Furthermore, the CR group registered higher plausibility ratings than the three OLP groups (active: *t*(179) = 4.12, *p* = <0.001, *d* = 0.86; usual: *t*(179) = 5.31, *p* = <0.001, *d* = 1.14; imaginary: *t*(179) = 2.69, *p* = 0.03, *d* = 0.59) (Figure 2). Other post hoc comparisons between groups were not significant (active OLP vs. usual OLP: *t*(179) = 1.18, *p* = 0.29, *d* = 0.24; active OLP vs. imaginary OLP: *t*(179) = 1.47, *p* = 0.29, *d* = 0.30).

## 4. Discussion

The present study investigated the effects of three different types of administering OLPs that varied in the number of motor actions involved in the intake of the placebo. The active OLP group was asked to complete a sequence of motor tasks before oral placebo intake, whereas the usual OLP group swallowed the unpacked pill without having received specific motor instructions. The imaginary OLP group did not have to carry out any motor actions (i.e., no actual intake of the placebo pill) before viewing the negative and neutral images. The three OLP interventions were compared with cognitive reappraisal (CR).

CR and the three OLP interventions were associated with similar valence ratings and physiological indicators (blood pressure, pulse). However, CR was accompanied by lower arousal compared to the imaginary OLP, regardless of picture type (negative, neutral). Since CR is an emotion-regulating strategy that is used in everyday life [24,25], this finding may be attributed to participants’ greater familiarity with the CR approach (as opposed to the imagination of taking a placebo). Moreover, in a previous study [26], participants indicated that the chosen CR instruction (‘This is not real’) was very easy to implement for reducing emotional distress. Following this view, participants registered higher ratings for the efficacy of CR than OLP. This replicates a previous finding of a study that directly compared CR and OLP interventions [26].

Surprisingly, the three OLP groups did not differ in their valence ratings during the viewing of the pictures. Increasing the emphasis on the motor components of the placebo intake did not increase the efficacy of the OLP. The present findings are not in line with the assumption that the physical action of taking placebo pills is a key active component of the mechanism that mediates placebo effects [7,8]. In the present study, even the ‘imaginary placebo’ was found to reduce emotional distress. Participants also reported higher plausibility ratings for this type of treatment, despite not consuming any pill, compared to the usual OLP group that did ingest a pill. This replicates the findings from a previous study [15]. However, the imaginary OLP group was characterized by the highest arousal rating during picture viewing (significantly higher than the CR group). Thus, the rating pattern lacked complete coherence. Nonetheless, it is important to highlight that all four groups reported very low arousal levels (M < 25, on a scale ranging from 0 to 100).

The present study has some limitations. First, we investigated an exclusively female sample with a predominant number of university students. Therefore, our results cannot be generalized to male participants and other genders. Second, the negative pictures were perceived as not being very intense. To elicit stronger psychophysiological responses, other types of stimulations (e.g., stress tests) could be selected for future studies. Third, the expectations associated with the interventions could have been influenced by the instructions that accompanied the distinct motor engagements. Following this hypothesis, it could have been informative to assess treatment-related expectations after the administration of general instructions (that did not address motor components of the treatment) and again after the administration of specific instructions that differed concerning the required motor actions. Fourth, the investigation of a clinical group should be considered. The efficacy of a placebo may differ between patients with diagnoses of mental/somatic disease versus healthy controls. For example, it has been shown that the placebo effect in the treatment of depression (severe emotional distress) is substantial [27]. Finally, the ’imaginary OLP’ condition was very likely not free of motor components. Some participants may have engaged in visualizing the act of taking a pill, thereby employing motor imagery.

In conclusion, this study demonstrated that reducing emotional distress with OLPs in healthy participants does not necessitate the consumption of a placebo pill. Not swallowing an OLP pill (and just imagining the intake) was even perceived as a more plausible intervention than swallowing it. The higher plausibility and efficacy ratings of the imaginary OLP group were, however, not accompanied by greater placebo effects. In terms of acceptability and ease of implementation, CR stands as a well-established alternative for reducing emotional distress.

## Figures and Tables

**Table 1:** Valence and arousal ratings (means, standard deviations).

Measure		Overall	Active OLP	Usual OLP	Imaginary OLP	CR
*M* (*SD*)		(*n* = 183)	(*n* = 45)	(*n* = 45)	(*n* = 47)	(*n* = 46)
**Valence**						
Baseline		9.6 (14.3)	9.8 (18.2)	9.8 (11.4)	9.5 (10.7)	9.4 (16.3)
Pictures	Overall	20.8 (12.8)	21.2 (13.1)	20.1 (13.7)	24.3 (12.4)	17.4 (11.6)
	Negative	35.7 (22.2)	38.0 (25.0)	34.6 (23.8)	40.1 (19.1)	30.0 (20.0)
	Neutral	5.9 (7.2)	4.3 (4.7)	5.6 (6.5)	8.5 (9.9)	4.9 (5.9)
**Arousal**						
Baseline		21.0 (19.4)	21.4 (19.7)	22.7 (19.8)	23.3 (18.6)	16.5 (19.6)
Pictures	Overall	15.8 (11.7)	15.8 (14.2)	14.7 (11.7)	19.8 (11.1)	13.0 (8.7)
	Negative	26.1 (19.2)	27.7 (24.7)	23.7 (18.6)	30.6 (16.3)	22.2 (15.7)
	Neutral	5.6 (7.4)	3.9 (5.8)	5.6 (7.5)	9.0 (10.1)	3.7 (3.2)

OLP: open-label placebo; CR: cognitive reappraisal.

## Data Availability

Data will be provided by the corresponding author upon request.

## Supplementary Materials

The following supporting information can be downloaded at: https://www.mdpi.com/article/10.3390/bs14060455/s1.
