# Supplementary material for: Reducing Emotional Distress with Open-Label Placebos: Assessing the Role of Motor Engagement in Pill Consumption
Source: Behav Sci (Basel). 2024 May 29;14(6):455. doi: 10.3390/bs14060455 (PMC11200763; doi:10.3390/bs14060455)
Supplement: Supplementary file 1 [file behavsci-14-00455-s001.zip › behavsci-2975629-supplementary.pdf]

Supplementary Material S1: Consort flow diagram

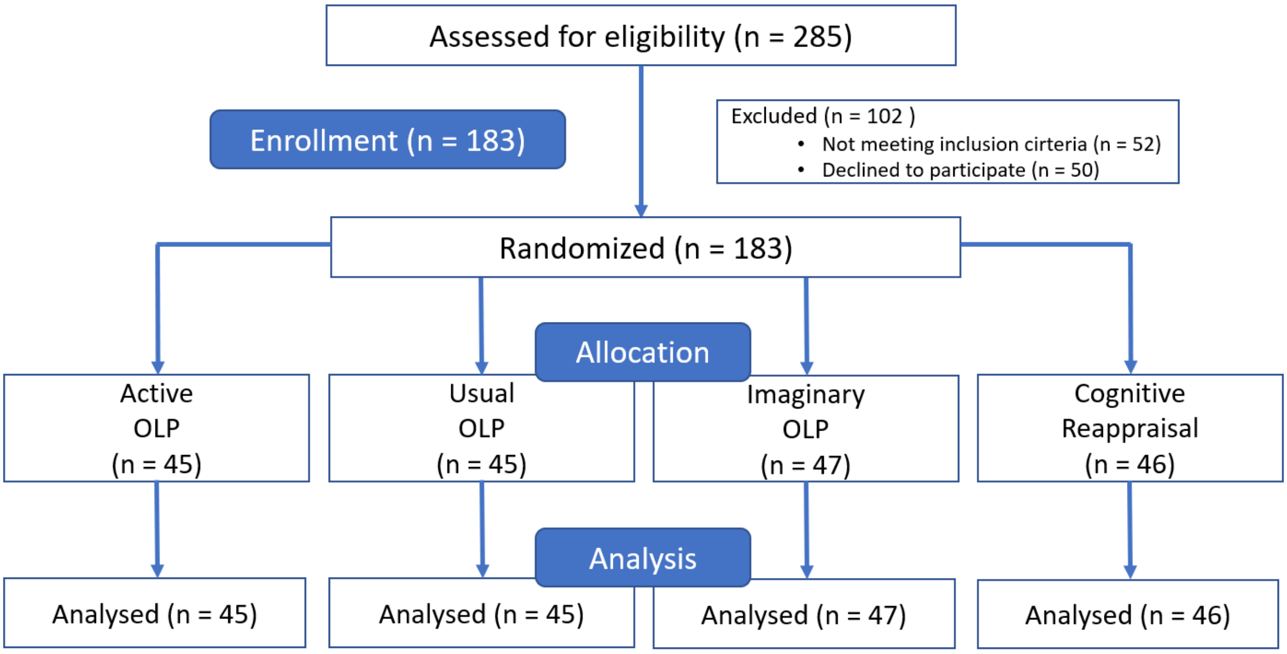

Supplementary Material S2. Pulse, systolic, and diastolic blood pressure (means, standard deviations) at baseline (T1), after specific placebo instruction/before picture viewing (T2), and after picture viewing (T3).

**Measure**	**Overall**	**Active OLP**	**Usual OLP**	**Imaginary OLP**	**CR**
M (SD)	**(*n* = 183)**	**(*n* = 45)**	**(*n* = 45)**	**(*n* = 47)**	**(*n* = 46)**
**Pulse (bpm)**					
T1	76.9 (13.9)	77.5 (12.5)	78.0 (17.0)	77.4 (13.0)	74.9 (12.9)
T2	76.6 (12.3)	79.2 (11.3)	74.8 (13.6)	76.6 (11.5)	75.7 (12.7)
T3	75.5 (11.8)	76.3 (11.6)	75.5 (13.3)	75.6 (11.1)	74.7 (11.3)
**Systolic BP (mmHg)**					
T1	133.0 (16.3)	133.5 (17.6)	135.2 (17.6)	134.5 (15.5)	130.9 (14.6)
T2	131.0 (14.5)	129.7 (14.0)	133.9 (14.8)	132.4 (16.6)	127.9 (11.8)
T3	132.0 (17.5)	131.8 (14.8)	133.0 (15.1)	132.7 (17.2)	129.0 (22.3)
**Diastolic BP (mmHg)**					
T1	84.4 (13.5)	84.0 (12.1)	85.7 (14.7)	85.0 (14.8)	83.1 (12.4)
T2	82.7 (11.7)	82.0 (10.8)	83.6 (11.4)	83.4 (14.4)	81.5 (10.0]
T3	83.8 (12.1)	83.7 (10.7)	84.3 (11.7)	83.6 (14.8)	83.6 (11.2)

Footnote: BP (blood pressure); OLP (Open-Label Placebo); CR (Cognitive Reappraisal)

Supplementary Material S3: Results of the ANOVAs for pulse and blood pressure (systolic, diastolic)

The ANOVA for pulse showed no significant effects for Group (F(3,179) = 0.51, p = .67, $\eta_p^2 = 0.01$) and Time (F(1,179) = 2.78, p = .10, $\eta_p^2 = 0.02$) , and a non-significant interaction Group x Time (F(3,179) = 1.37, p = .25, $\eta_p^2 = 0.02$).
The ANOVA for systolic blood pressure revealed non-significant effects for Group (F(3,179) = 1.07, p = .37, $\eta_p^2 = 0.02$), Time (F(1,179) = 0.43, p = .52, $\eta_p^2 = 0.02$) and Group x Time (F(3,179) = 0.40, p = .75, $\eta_p^2 = 0.01$).
The ANOVA for diastolic blood pressure revealed no significant effects for Group (F(3,179) = 0.13, p = .94, $\eta_p^2 = 0.002$) and Group x Time (F(3,179) = 0.56, p = .64, $\eta_p^2 = 0.01$). The effect for Time was statistically significant (F(1,179) = 3.94, p = .049, $\eta_p^2 = 0.02$). Diastolic blood pressure was higher after the picture viewing than before (see supplementary Material S2).
